# Fiscal transfers and public health services in China: a macro–micro analysis

**DOI:** 10.3389/fpubh.2025.1577963

**Published:** 2025-04-09

**Authors:** Xunhua Tu, Jie Yan, Yueyi Wang, Jing Zheng

**Affiliations:** ^1^School of Economics, Sichuan University of Science & Engineering, Zigong, China; ^2^School of Public Finance and Taxation, Southwestern University of Finance and Economics, Chengdu, China; ^3^Sichuan Lanxin Urban and Rural Community Construction Research Center, Chengdu, China; ^4^School of Economics and Management, Sichuan Polytechnic University, Deyang, China

**Keywords:** fiscal transfers, public health service levels, fiscal spending bias, fiscal health spending efficiency, fiscal gap

## Abstract

**Introduction:**

This study innovatively integrates functional performance and systemic capacity dimensions to establish a comprehensive evaluation framework for public health services, addressing critical knowledge gaps in fiscal policy-health system interactions.

**Methods:**

Utilizing multi-source spatial panel data from 1,871 Chinese counties (2001-2020), we developed a novel assessment system combining structural equation modeling (SEM) and fuzzy comprehensive evaluation. Complementary analyses of 283 prefecture-level cities employed fixed-effects models with instrumental variables to examine fiscal transfer mechanisms.

**Results:**

Our findings reveal: Significant positive association between fiscal transfers and service levels. Dual-path mediation effects: Fiscal expenditure restructuring; Health spending efficiency optimization. Micro-level health equity improvements.

**Discussion:**

These evidence-based results provide policymakers with actionable insights for: Establishing performance-oriented accountability frameworks; Promoting health equity through fiscal system optimization; The proposed multidimensional evaluation system offers a replicable toolkit for global health governance assessment.

## 1 Introduction

Health serves both as a means for people to make a living and as a guarantee that allows them to enjoy the fruits of their labor (1). Public health services are crucial government-provided public services that safeguard and enhance societal health and improve residents’ quality of life. Particularly in the context of the current global health crisis and frequent public health emergencies, public health services have become a key pillar for the stable development of the national economy and society. As a vast and populous developing country, China still faces significant regional disparities in public health service levels. Data from 2021 show that the ratio of per capita healthcare expenditures between the highest and lowest provinces is as high as 3.34, and the ratios of healthcare technicians and hospital beds per 10,000 people are 1.9 and 36.61, respectively, highlighting welfare disparities due to unequal resource distribution.

The impact of fiscal transfers, as a crucial policy tool for achieving social equity and enhancing public health services, has increasingly attracted attention from academics and policymakers (2–4). In China, given the significant disparities in regional economic development, the current transfer payment system remains the central government’s primary means to increase public goods supply under existing constraints, making fiscal transfer payments particularly significant.

With China’s rapid economic development, the scale of fiscal transfers has expanded, providing more funds for local governments to improve basic medical facilities and public health services, contributing to the equalization of public health services across provinces (5–7). Additionally, transfer payments help improve population health (8), which serves not only as an outcome of economic development but also as a foundation for social stability and progress. Therefore, in the context of China, studying the impact of fiscal transfers on public health service welfare can offer policymakers an effective basis for decision-making and an important reference for achieving the goal of universal health coverage.

Although studies have explored the impact of fiscal transfers on public health, the mechanisms underlying their role in the specific context of China still require in-depth analysis. Factors such as fiscal expenditure efficiency, expenditure priorities, and regional disparities in financial resources all influence the final level of public health services. Moreover, given China’s unique economic and social context, determining how to effectively improve the quality of public health services and reduce health inequalities through fiscal policies remains a crucial issue to be addressed.

The purpose of this paper is to examine whether fiscal transfers impact the welfare of public health services. We analyze the pathways through which fiscal transfers influence public health services and their effect on residents’ health welfare from both macro and micro perspectives, exploring in detail how fiscal means can narrow health inequality and achieve the goal of universal health. To achieve these results, we integrate Sen’s capability approach to develop an evaluation system for public health service indicators from a functional and capacity perspective. We measure public health service levels using data from 1,871 districts and counties in China from 2001 to 2020, applying structural equation modeling and fuzzy evaluation methods. Based on panel data from 283 prefecture-level cities in China from 2001 to 2020, we conduct empirical tests using a two-way fixed-effects model and instrumental variables approach. Our results show that fiscal transfers enhance public health service levels, with no non-linear relationship observed. Moreover, at the 1% significance level, a 1% increase in transfer payments leads to a 0.007% improvement in public health services.

We address the potential endogeneity between transfers and the level of public health services using an instrumental variables approach. To further validate our findings, we conduct a series of robustness tests, including replacing the explanatory variables’ measures and using principal component analysis to re-measure public health service levels as an explanatory variable for re-regression. Additionally, we conduct robustness tests by excluding samples with significant economic and social differences, such as municipalities, adjusting the sample duration, and reducing the sample size. The results confirm that the baseline regression findings are robust, indicating that the impact of fiscal transfers on enhancing public health service levels is substantial and not merely a statistical coincidence due to specific measurement methods, sample selection, or time periods.

Our heterogeneity analysis shows that fiscal transfers do not significantly impact public health service levels in the eastern region, whereas they have a significant positive impact in the central and western regions compared to the eastern part of the country. This indicates that the central and western regions receive greater support from transfer payments, providing them with more financial resources to improve public health services, which has a greater effect on service levels. Additionally, we examine the heterogeneous impact of different types of transfers. Compared to general transfers, specialized transfers are more effective in improving public health service levels.

In further discussing the impact of transfers on residents’ micro health, we draw on data from the China Health and Retirement Longitudinal Study (CHARLS), a high-quality micro dataset representative of Chinese households and individuals aged 45 years and older. The CHARLS questionnaire design is based on international surveys, including the US Health and Retirement Survey (HRS), the UK Tracking of Ageing Survey (ELSA), and the Survey of Health, Ageing and Retirement in Europe (SHARE). It includes basic personal information, family structure and financial support, health status, physical measurements, health care utilization and health insurance, work, retirement and pension, income, consumption, assets, and community information. The project used multi-stage sampling with PPS sampling methods at both district and village levels. Our estimates suggest that transfers help reduce inequalities in public health services and improve population health at the micro level. Each 1% increase in transfer payments results in a 0.013% decrease in public health service inequality. Based on these findings, we argue that fiscal transfers can ultimately improve population health by reducing inequalities in public health services.

We then explore the first potential mechanism by which transfers may affect the level of public health services. Consistent with the findings of Ding and Zhang (9), we observe that local governments receiving transfer payments are more inclined to increase the share of welfare expenditures within productive expenditures, thereby raising local government investment in welfare. The significant impact of fiscal expenditure priorities on improving public health services suggests that active financial inputs can optimize resource allocation, quality, and coverage of public health services in localities, thus contributing to their overall improvement.

A second potential factor driving our findings is the increased efficiency of fiscal health expenditures by local governments as transfers grow. Improved fiscal health expenditure efficiency, in turn, affects the allocation of resources, quality, coverage, and the persistence of inequalities in public health services across regions. Our evidence indicates that higher fiscal health expenditure efficiency enhances public health service levels.

Our study also emphasizes the role of the fiscal gap. While the original purpose of transfers was to reduce the fiscal gap between regions, empirical results indicate that transfers actually widen the fiscal gap between local governments, consistent with the findings of Wang and Tao (10). Thus, this mechanism does not function as intended in practice.

Compared to existing studies, this paper offers three main innovations: First, it directly contributes to enriching the current research on how transfer payments affect public health service levels. Our study examines the impact of transfers at the city level, addressing endogeneity using the instrumental variables approach, and provides strong evidence for a causal relationship between fiscal transfers and public health service levels in China. Second, we explored the state of residents’ public health services from both functional and capacity perspectives, employing structural equation modeling and a fuzzy evaluation method to measure public health service levels in China. This approach provides a more comprehensive and realistic reflection of residents’ welfare. Finally, our paper extends the existing literature on the impact of fiscal transfers on health inequality at the micro level. We not only identify the effect of fiscal transfers on public health service levels but also examine their influence on micro-level health inequality among residents.

The rest of the paper is structured as follows: Section 2 summarizes the background of transfer payment policies. Section 3 describes the data sources and presents the empirical research design. Section 4 examines the impact of fiscal transfers on public health service levels, including heterogeneity effects, and verifies the robustness of the empirical results. Section 5 uses microdata to analyze the impact of fiscal transfers on health inequality, further extending the analysis. Section 6 explores the possible mechanisms through which transfer payments affect public health services. Finally, the paper concludes with policy recommendations.

## 2 Institutional context

In 1994, China implemented a reform of the tax-sharing system, which established a hierarchical budgetary management structure dividing central and local fiscal revenues based on tax types and responsibilities. This reform clarified the allocation of central and local tax revenues and laid the foundation for a transfer payment system. Following the tax-based financial system reform, a transfer payment system comprising institutional subsidies, special subsidies, and tax refunds was initially established. Through fiscal transfers, the central government provides financial resources to local governments to meet their expenditure needs, particularly in infrastructure, education, and healthcare.

Before 2005, the central government’s transfer payments to localities were primarily divided into tax refunds and various subsidized expenditures. From 2006 to 2018, China’s intergovernmental transfer payments mainly comprised three categories: general transfer payments, special transfer payments, and tax refunds. After 2019, China adjusted the structure of its transfer payments by merging tax refunds into general transfer payments. Currently, China’s transfer payments mainly include general and special transfer payments, which accounted for 63.82 and 6.27% of the central general public budget expenditures in 2021, respectively (as measured by the Ministry of Finance of China). General transfers are primarily intended to balance disparities in local financial resources without specific usage targets, ensuring the normal operation of basic public services. Local governments have significant autonomy in deciding how to allocate these transfers, allowing local preferences to influence the use of funds. Economic preferences may lead to increased productive expenditures, such as infrastructure development, poverty reduction, and employment promotion, thereby enhancing residents’ welfare. Livelihood preferences, on the other hand, focus on welfare expenditures, such as education, healthcare, and social security, which have a greater impact on improving overall welfare. Specialized transfers are designated for specific policy objectives, such as education and healthcare, with defined conditions and usage purposes. They can redirect government fiscal expenditures and improve infrastructure or public services in less developed regions.

**Figure 1:**
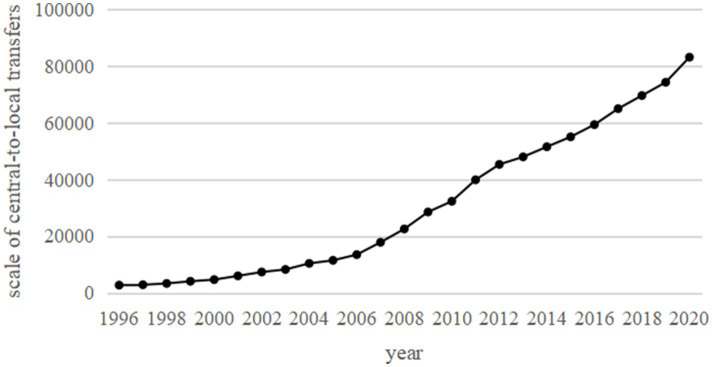
Scale of central-to-local transfers, 1996–2020 unit: billion yuan.

Since the 1994 reform of the fiscal tax-sharing system, the scale of central-to-local transfers has continued to grow. This growth has responded both to economic development and to local financial needs in areas such as social security, infrastructure construction, and others. Figure 1 shows the trend of China’s central-to-local transfer payments from 1996 to 2020 (Note: the classification of transfers was adjusted during the sample period; here, transfers are presented as the sum of tax rebates and transfers to ensure consistency. Data are from the EPS database, fiscal yearbooks, and the Ministry of Finance website). Over the past decades, the central government has consistently increased transfer payments to localities, resulting in a significant overall growth trend. Notably, the scale of transfer payments has risen sharply since 2007, likely related to China’s rapid economic growth and the government’s proactive fiscal adjustments. The increase in transfer payments was particularly notable during the economic crisis of 2008, reflecting the central government’s response to dynamic macroeconomic and social needs through fiscal measures. By 2020, the scale of central-to-local transfer payments reached 8,321.8 billion yuan, nearly 30 times the level in 1996, accounting for 70.34% of the central government’s fiscal expenditures.

## 3 Data and empirical research design

### 3.1 Data sources and processing

In this section, panel data from prefecture-level and above cities from 2001 to 2020 are selected for empirical analysis. Data such as fiscal transfer payments are primarily sourced from the National Statistics of Prefecture, Municipality, and County Finances, the China Fiscal Statistical Yearbook, provincial statistical yearbooks, and provincial fiscal final accounts, among others. Other data are obtained from provincial and city statistical yearbooks, the China Urban Statistical Yearbook, the EPS database, and the CEIC economic database. The data processing in this paper includes the following steps: First, samples with missing key variables, such as fiscal transfer payments, are excluded. Second, 2001 is set as the base year, and nominal variables are adjusted for inflation. Third, natural logarithms are applied to variables such as fixed asset investment and per capita fiscal expenditure to ensure that the data approximate a normal distribution. As a result, we obtain panel data for 283 prefecture-level and above cities from 2001 to 2020, totaling 5,660 samples.

### 3.2 Research methodology and study design

To address the questions raised in the previous analysis and examine the impact of transfers on public health service levels, this section constructs the following two-way fixed effects benchmark model:


(1) $$Heal_{\mathrm{it}}=\beta_{0}+\beta_{1}\mathrm{Lntra}n_{\mathrm{it}}+\beta_{2}X_{\mathrm{it}}+\alpha_{i}+\mu_{\mathrm{pt}}+\varepsilon_{\mathrm{it}}$$

In Equation 1, i represents the city, and t represents the time, and $Heal_{\mathrm{it}}$ denotes the level of public health services in city i during year t . $\mathrm{Lntra}n_{\mathrm{it}}$ indicates the size of per capita transfer payments in city i during year t . $\beta_{1}$ represents the impact of fiscal transfers on public health service levels, which is the focus of this section. $X_{\mathrm{it}}$ is a series of control variables. $\alpha_{i}$ denotes city fixed effects, and $\mu_{\mathrm{pt}}$ represents the interaction term between province fixed effects and year fixed effects. $\beta_{0}$ is a constant term, and $\varepsilon_{\mathrm{it}}$ denotes the random perturbation term.

Since transfer payments encourage the expansion of productive expenditures in county-level finances, they fail to incentivize local governments to improve the provision of social public goods. Moreover, expenditure subsidies beyond a certain threshold significantly reduce the level of social public goods supplied by local governments (11). This indicates that the effect of transfer payments on the structural bias of basic public services exhibits an “inverted U-shaped” relationship (12). To test whether a non-linear relationship exists between transfer payments and the level of public health services, this section further investigates the relationship using the threshold effect regression model proposed by Hansen (34) in the form of a panel threshold model, as follows:


(2) $$\begin{array}{l} Heal_{\mathrm{it}}=\beta_{0}+\beta_{1}\mathrm{Lntra}n_{\mathrm{it}}I\left(\mathrm{Lntra}n_{\mathrm{it}}\leq \sigma_{1}\right)+\beta_{2}\mathrm{Lntra}n_{\mathrm{it}}I\\ \;\left(\sigma_{1}<\mathrm{Lntra}n_{\mathrm{it}}\leq \sigma_{2}\right)+\beta_{3}\mathrm{Lntra}n_{\mathrm{it}}I\left(\mathrm{Lntra}n_{\mathrm{it}}>\sigma_{2}\right)\\ \;+\theta X_{\mathrm{it}}+\varepsilon_{\mathrm{it}} \end{array}$$

In Equation 2, $Heal_{\mathrm{it}}$ denotes the level of public health services in city i during year t . $\mathrm{Lntra}n_{\mathrm{it}}$ indicates the size of per capita transfer payments in city i during year t . The size of per capita transfers in year t is also the threshold variable used in this section. $\sigma_{i}$ represents the threshold value to be estimated. $I\;\left(\cdot \right)$ is the indicator function, which takes the value of 1 if the condition in parentheses is satisfied, and 0 otherwise. $\beta_{1},\beta_{2},\beta_{3}$ are the coefficients representing the influence of the explanatory variable $\mathrm{Lntra}n_{\mathrm{it}}$ on the explained variable $Heal_{\mathrm{it}}$ . $X_{\mathrm{it}}$ is a set of control variables. $\beta_{0}$ is the constant term, and $\varepsilon_{\mathrm{it}}$ denotes the random perturbation term.

### 3.3 Main variables and descriptive statistics

#### 3.3.1 Main variables

(1) Explained variable: level of Public Health Services (*heal*). Based on the 14th Five-Year Plan and the 20th National Congress Report, and drawing on existing literature (13–15, 32, 35), this paper constructs an indicator evaluation system for the level of public health services in terms of function and capacity. “Function” reflects the actual effect of government policies and the quality of life of residents, while “capacity” represents the choices and opportunities provided by the government to residents. Specific indicators and definitions are presented in Table 1.

**Table 1:** Indicator evaluation system for the level of public health services.

Level 1 indicators	Secondary indicators
Functional indicators	Mortality rate (‰) [−]
	Health facilities per 10,000 population [+]
	Medical beds per 10,000 population [+]
	Health technicians per 10,000 population [+]
Capacity indicators	Percentage of local government expenditure on health care [+]
	Financial health expenditure per capita [+]

The direction of the indicator is given in [].

This paper employs a combination of structural equation modeling and the fuzzy evaluation method to assess the level of public health services among China’s residents, offering several advantages. Firstly, structural equation modeling enables the examination of relationships between indicators across various welfare dimensions and the overall welfare level, ensuring greater accuracy and objectivity in computing indicator weights based on relationship coefficients. Secondly, structural equation modeling facilitates the assessment of relationships between specific indicators and latent variables through the factor loading matrix, allowing for the evaluation of the extent to which latent variables are represented by selected indicators. Thirdly, the fuzzy evaluation method has a better stability, enabling the reasonable evaluation and measurement of residents’ welfare levels. The integration of these two methods allows for a more reasonable and accurate measurement of China’s welfare level.

The main measurement idea of this paper is: First, using the welfare model, the fuzzy evaluation method [some scholars have used the fuzzy comprehensive evaluation method to measure welfare levels based on fuzzy mathematics created by Zadeh (14, 15, 36–38)] is applied to determine the degree of affiliation of each indicator to welfare and to obtain the fuzzy evaluation matrix. Second, the structural equation model [first introduced by Jöreskog (39), further refined by Jöreskog and Goldberger (40), initially utilized in psychology and sociology by Bentler and Weeks (41), and later used in economic research by Raiser and Weeks (42); also utilized by Kuklys (43), Krishnakumar and Ballon (44), and De Rosa (13) in competency assessment] is used to test the extent to which each indicator captures each functional dimension, setting the weights of each indicator based on the correlation coefficient. Finally, referring to the summing method of Cerioli and Zani (16) and Yuan and Shi (15), the degrees of affiliation are summed to obtain the public health service level.

(2) Core explanatory variables: size of *Per Capita* Transfers in Each Region (*lntran*). The logarithm of per capita transfer size in each region is used as a proxy variable. Transfers include general transfers, specialized transfers, and tax rebates.

(3) Mechanism variables: Fiscal Expenditure Bias (*exp*), Fiscal Health Expenditure Efficiency (*heff*), and Fiscal Gap (*fgap*) across municipalities. Fiscal expenditure bias is represented by the ratio of welfare fiscal expenditure to total fiscal expenditure (*wfisex*) relative to the ratio of productive fiscal expenditure to total fiscal expenditure (*pfisex*) ( $\exp=\frac{\mathrm{wfisex}}{\mathrm{pfisex}}$ ). Since, after the reform of fiscal subjects in 2007, original capital expenditures were categorized within each subject class based on functional classification, this paper follows existing theories and related literature (17) for expenditure classification before and after 2007. Before 2007, productive expenditures were represented by capital expenditures and agricultural, forestry, and water expenditures, while welfare expenditures were represented by spending on education, social security, and healthcare. After 2007, productive expenditures are primarily economic construction expenditures excluding government function expenditures, social, cultural, and educational expenditures, and other expenditures, while welfare expenditures primarily include spending on education, science and technology, culture and sports, social security and employment, and medical and healthcare.

The efficiency of fiscal health expenditure is measured using the Cobb–Douglas form of a cost-based stochastic frontier model, employing the stochastic frontier analysis (SFA) approach as described by Tang and Wang (18) and Xu et al. (19):


(3) $$\ln\mathrm{hfie}x_{\mathrm{it}}=\alpha_{0}+\overset{N}{\underset{j=1}{\sum }}\alpha_{j}\ln PS_{ijt}+v_{\mathrm{it}}+u_{\mathrm{it}}$$

In Equation 3, $\mathrm{hfie}x_{\mathrm{it}}$ represents the fiscal health expenditure of area i in year t (measured here as local government per capita health expenditure), and $PS_{ijt}$ denotes the level of supply of public service j in year t in region i . As the focus here is on measuring the efficiency of fiscal expenditure in public health, indicators of public goods supply, such as healthcare, are primarily used as output variables to assess the efficiency of fiscal health expenditure by local governments. The specific indicators are shown in Table 2. $v_{\mathrm{it}}$ is the random error term, and $u_{\mathrm{it}}$ denotes the null term. The fiscal gap (*fgap*), on the other hand, draws on Zhao and Fu (33) and is measured using the per capita value of each city’s financial power in comparison to the mean value of the province in which the city is located. A smaller fgap value indicates that the change in regional financial power is closer to the average level and is converging. The formula for fiscal gap is given as: fgap = fisex_ipt_/fisex_pt_, where fisex_ipt_ denotes the per capita fiscal expenditure of city *i* in province *p* during year *t*, and fisex_pt_ is the average value of fiscal expenditure in province *p*.

**Table 2:** Output indicators of local government public goods provision for health care.

Level 1 indicator	Secondary output indicators
Medical care	Hospitals per 10,000 population
	Medical beds per 10,000 population
	Doctors per 10,000 population

(4) Control variables: the level of economic development may be related to public health services, as more developed regions usually have more resources and can provide better medical facilities and services. Investment in fixed assets may reflect the construction of infrastructure and healthcare facilities in a region. Regions with good infrastructure are more likely to provide better public health services. Additionally, demographic differences may affect the demand for public health services. The level of regional openness to the outside world may also be associated with economic development and resource allocation, which can influence the provision and quality of public health services. Therefore, controlling for these factors allows for a more accurate assessment of the independent impact of transfers on public health service levels. This section controls for the level of economic development (*lngdp*), fixed asset investment (*lnasset*), population structure (*pop*), and regional openness (*fdi*).

#### 3.3.2 Descriptive statistics

The descriptive statistics of the main variables are presented in Table 3, showing a significant gap between the minimum and maximum values of each variable.

**Table 3:** Variable definitions and descriptive statistics.

Variable	Variable definition	Observed value	Average value	Standard deviation	Min value	Max values
*Heal*	Combined level of functioning and capacity of public health service dimensions	5,660	0.369	0.180	0.017	0.951
*lntran*	The size of per capita transfers by region is taken in logarithms	5,657	7.371	1.162	3.446	11.988
*lngdp*	Logarithmic GDP per capita by region	5,660	10.203	1.282	6.743	18.127
*lnasset*	Investment in fixed assets by region in logarithmic terms	5,659	9.468	1.343	6.422	11.665
*pop*	natural population growth rate	5,660	5.495	4.498	−5.6	19.88
*fdi*	Foreign direct investment as a share of GDP	5,660	0.019	0.023	0	0.476
*exp*	$\exp=\frac{\mathrm{wfisex}}{\mathrm{pfisex}}$	5,660	1.075	2.398	0.035	6.340
*heff*	Efficiency of financial health expenditures by region	5,660	0.591	0.155	0.085	0.876
*fgap*	Disparity of financial resources among regions	5,660	0.040	0.712	−0.975	8.728

## 4 Analysis of empirical results

### 4.1 Benchmark regression results

In this section, based on panel data from 283 prefecture-level and above cities in China from 2001 to 2020, a two-way fixed-effects model is selected for regression analysis. This model simultaneously controls for individual characteristics that do not vary over time and general time effects, thus providing a more accurate estimation of the impact of transfer payments on public health service levels. Additionally, robust standard errors clustered at the city level are used in the estimation process to account for potential heteroskedasticity, cross-sectional correlation, and time-series correlation. The regression results of transfer payments on public health service levels are presented in Table 4. Columns (1)–(5) show the regression results for different scenarios: without control variables or fixed effects, with control variables only, controlling for individual and year effects only, adding control variables while controlling for individual and year fixed effects, and adding control variables while controlling for individual as well as province-year interaction fixed effects. Comparing the results across these columns, all fiscal transfers are found to have a significant positive effect on public health service levels, suggesting that transfers help improve public health services for residents. This implies that the role of transfer payments in enhancing public health services aligns with their objective of promoting public service equalization. By increasing transfers to impoverished or under-resourced areas, the government can effectively target the improvement of public health services in these areas, thereby raising the overall level of health services.

**Table 4:** Impact of transfer payments on public health service levels.

Variable	Heal				
	(1)	(2)	(3)	(4)	(5)
*Lntran*	0.017^***^ (0.004)	0.013^***^ (0.005)	0.011^***^ (0.004)	0.008^**^ (0.004)	0.007^***^ (0.003)
*_cons*	0.245^***^ (0.032)	0.200^***^ (0.045)	0.284^***^ (0.024)	0.294^***^ (0.025)	0.334^***^ (0.022)
Control variable	NO	YES	NO	YES	YES
City fixed effect	NO	NO	YES	YES	YES
Year fixed effects	NO	NO	YES	YES	NO
Province × Year fixed effects	NO	NO	NO	NO	YES
Number of observations	5,657	5,657	5,657	5,657	5,657
*R*-squared	0.126	0.154	0.126	0.080	0.045

*** and ** represent significant at the 1 and 5% level, respectively, and robust standard errors clustered to the city level are in parentheses.

To test the possible non-linear relationship between transfer payments and public health service levels, this section estimates the relationship using a panel threshold effects model. The panel threshold effects model is an effective tool for analyzing non-linear relationships between variables and can reveal the pattern of changes in public health service levels influenced by different levels of transfer payments. Before conducting the threshold effect analysis, the actual number of thresholds in the panel data is determined. In this section, single- and double-threshold tests are conducted on the threshold variable of transfer payments, with 500 random samples drawn from the threshold variable until the corresponding threshold effect is found to be insignificant. This process identifies whether there are one or more shifting points in the data, which are critical in understanding the impact of transfer payments on public health service levels. The specific test results are presented in Table 5. The single-threshold test for transfers passes the significance test at the 1% level, suggesting that a transition point may exist between transfers and public health service levels. This transition point may indicate that the intensity and direction of the impact of transfer payments on public health service levels vary significantly at different levels of transfer payments. It also suggests that there may be an optimal level of transfers, above or below which the effect on public health service levels differs. This means that rather than focusing solely on the volume of fiscal transfers, attention should also be given to the efficiency and effectiveness of their allocation. Specifically, fiscal transfers may be more effective in regions or sectors that are underfunded or have lower fiscal self-sufficiency, but beyond a certain threshold, the marginal benefit of additional transfers may be reduced.

**Table 5:** Threshold effect test results of transfer payments on public health service levels.

Threshold variables	Threshold test	Threshold value	*F*-value	P-value	10% threshold	5% threshold	1% threshold
*Lntran*	Single Threshold	8.801	118.75	0.002	70.039	81.045	98.024
	Double Threshold	8.801	118.75	0.002	68.841	78.190	99.271
		6.779	31.33	0.310	48.053	55.898	72.281

### 4.2 Analysis of endogeneity

Transfer payments from the central government provide local governments with additional financial resources that can be used to improve public services, including health and medical services. If transfers are directed toward disadvantaged areas or groups, they may enhance health service levels in those areas or groups. However, if significant gaps exist in local public health services and low levels of well-being are observed—meaning that certain areas or groups lack access to basic healthcare—these local governments may require more transfers to close these gaps. Consequently, the level of local public health services may become an important factor for the central government when adjusting its transfer policy. Very low levels of local services may attract the central government’s attention, prompting it to adjust its transfer payment strategy and invest additional resources in health service improvements. In short, there may be reverse causality between fiscal transfers and public health service levels: increased fiscal transfers may help raise public health service levels, and, in turn, the level of local public health services may create increased demand for transfers, as the central government aims to raise service levels and address disparities by providing additional funds. To mitigate the endogeneity problem caused by potential reverse causality between the explained and explanatory variables or omitted variables, this section is estimated using the instrumental variables method.

This section uses two variables as instrumental variables. The first is the size of per capita transfers in the province where each city is located. Since the transfers received by each prefecture are determined by the size of transfers received by the province and are also influenced by higher-level government decisions, there is a correlation between the size of per capita transfers at the provincial level and the transfers received by the prefecture. Additionally, the transfers received by the provinces do not directly affect the level of public health services in the regions, making this variable consistent with exogeneity. Second, following Ma and Meng (20) and Nakamura and Steinsson (21), Bartik-style instrumental variables are constructed for regression. Specifically, the construction method uses the interaction term between the initial year’s ranking of each city’s level of development in the province (GDP per capita ranking in 2001) and the total amount of superior transfers received by each city in the province as the instrumental variable for each city’s transfers. Since the transfers received by each municipality are influenced by the size of transfers received by the province and the decisions of higher-level government, there is a correlation between provincial per capita transfers and municipal transfers. Moreover, the total amount of transfers received by municipalities does not directly impact the level of public health services in a specific municipality, making the instrumental variable largely satisfy the condition of exogeneity. Additionally, the initial year’s ranking is not affected by current transfers within the sample period. The initial level of economic development ranking, even if it has a direct effect on the current level of public health services, is accounted for by city fixed effects as an inherent city factor. Furthermore, if the initial economic development ranking has a time-varying effect on the current level of public health services, it has been controlled for by the interaction term between the logarithm of the city’s GDP per capita and the year dummy variable.

The instrumental variable regression model for the first stage is:


(4) $$\begin{array}{l} \ln\left(\mathrm{tran}s_{\mathrm{it}}\right)=\beta_{0}+\beta_{1}\mathrm{Ptran}s_{\mathrm{pt}}+\beta_{2}Ln\left(\mathrm{totra}n_{ipt}\times \mathrm{ran}k_{i}\right)\\ \;+\beta_{3}X_{\mathrm{it}}+\alpha_{i}+\varepsilon_{\mathrm{it}} \end{array}$$

In Equation 4, the $\mathrm{Ptran}s_{\mathrm{pt}}$ denotes the per capita transfer payment scale in province *p* where each city is located in year *t*. $\mathrm{totra}n_{ipt}$ represents the total transfer payments received in province *p*, where city *i* is located, in year *t*. $\mathrm{ran}k_{\mathrm{it}}$ denotes the per capita GDP ranking of city i in the province in 2001. The definitions of the remaining control variables are consistent with Equation 1.

The results of the regression using the 2SLS method are presented in Table 6. After accounting for endogeneity, the results are consistent with the basic regression, and financial transfer payments are still significantly negatively associated with inequality in public health services. Furthermore, in the test results for the non-identification of instrumental variables, the *p*-value of the Kleibergen-Paap rk LM statistic is 0.000, rejecting the null hypothesis of “under-identification of instrumental variables” at the 1% significance level. The F statistic of the first-stage regression is greater than 10, indicating that there is no problem of weak instrumental variables. These test results show that the selected instrumental variables have a negative relationship with inequality in public health services, consistent with the basic regression, and confirm the rationality of the instrumental variable selection.

**Table 6:** Instrumental variable regression results.

Variable	First-stage Regression	Second-stage Regression
	Lntran	Heal
	(1)	(2)
*IV1: Ptrans*	0.065^***^ (0.023)	
*IV2:* totran×rank	0.945^***^ (0.021)	
*Lntran*		0.011^***^ (0.004)
Control variable	YES	YES
City fixed effect	YES	YES
Number of observations	5,660	5,660
Kleibergen-Paap rk LM statistic		139.454 [0.0000]
*F*-value	1650.88	
*R*-squared	0.693	0.104

*** represents significant at the 1% level, values within () are robust standard errors for clustering to the city level. Values within [] are *p*-values.

### 4.3 Robustness analysis

To verify that the results of the study are consistent across different variables, methodological changes, or sample period adjustments, this section conducts the following robustness tests. First, the measurement method of the explanatory variables is replaced. To avoid unreliable regression results due to measurement errors, the explanatory variable “public health service level” is re-measured using principal component analysis, which serves as an explanatory variable in the regression. This method quantifies public health service levels from a different perspective and helps verify the reliability of the original measurement method. Second, some samples are excluded. Since municipalities may have significantly different economic and social characteristics compared to other cities, excluding these special samples allows us to determine if the effect of transfer payments on public health service levels remains significantly positive, suggesting that this effect exists across different types of cities. Third, the sample period is adjusted. Shortening the sample period to 2010–2020 tests whether the study results are influenced by special events or conditions during specific time periods. If the impact of transfers on public health service levels remains consistent, this indicates that the findings are robust over time. Fourth, the sample is trimmed. Trimming the data at the 1st and 99th quantiles for continuous variables removes the impact of extreme values on the study, improving the reliability of data analysis and the stability of the results. Finally, we use a dynamic panel model to examine lagged fiscal transfer effects.

The results of the robustness tests are presented in Table 7. After replacing the explanatory variables, excluding part of the sample, and adjusting the sample period, the coefficients of transfer payments and public health service levels remain significantly positive. This indicates that the results of the benchmark regression are robust, reflecting that the impact of fiscal transfers on enhancing public health service levels is substantial and not merely a result of a specific measurement methodology, sample selection, or time period. These findings support the view that transfer payments are an effective tool for reducing inter-regional disparities in public health services and upgrading health service levels, providing a basis for the formulation and implementation of related policies.

**Table 7:** Results of robustness tests.

Variable	Heal				
	(1) Replacement measurement method	(2) Excluding some samples	(3) Shorter sample period (2010–2020)	(4) Trim sample	(5) GMM
*Lntran*	0.004^**^ (0.002)	0.008^***^ (0.002)	0.012^**^ (0.005)	0.008^***^ (0.003)	0.027^***^ (0.001)
*_cons*	0.031^**^ (0.013)	0.320^***^ (0.021)	0.297^***^ (0.042)	0.322^***^ (0.022)	0.167^***^ (0.004)
Control variable	YES	YES	YES	YES	YES
City fixed effect	YES	YES	YES	YES	YES
Province × Year fixed effects	YES	YES	YES	YES	YES
Number of observations	5,656	5,577	3,112	5,638	5,660
*R*-squared	0.020	0.044	0.029	0.047	—

* and *** represent significant at the 10 and 1% levels, respectively, and robust standard errors clustered to the city level are in parentheses.

### 4.4 Heterogeneity analysis

#### 4.4.1 Impact of transfer payments on public health service levels in different regions

Differences in economic development levels across regions may lead to varying demands for public health services. Developed regions may have higher demands for health services, while less developed regions may require more financial support to improve infrastructure and healthcare service levels. The government has adopted different scales and modes of transfer payment strategies for different regions. For regions with poorer health services, the government may be more inclined to provide additional financial support to enhance health service levels. Therefore, the impact of transfer payments on public health service levels may vary across regions. In this section, the sample is divided into three regions: east, central, and west, and possible heterogeneous impacts of transfer payments are examined using group regression models.

The results of the test are presented in Table 8. Column (1) shows the regression results grouped by region. In the eastern region, the coefficient of financial transfers on public health service levels is negative and statistically insignificant, whereas the impact of transfers on public health service levels in the central and western regions is greater and more significant than in the east. This suggests that the increased transfer support received by the central and western regions provides more financial resources to improve public health services, leading to a greater impact in these regions.

**Table 8:** Impact of transfers on public health service levels by subcategories and subregions.

Variable	Heal		
	(1)	(2)	(3)
*Lntran*	−0.001 (0.001)		
*Area × Lntran*			
Central	0.003^***^ (0.001)		
Western	0.007^***^ (0.001)		
*Lngtrans*		0.003^*^ (0.002)	
*Lnstrans*			0.007^***^ (0.002)
*_cons*	0.046^***^ (0.009)	0.361^***^ (0.015)	0.343^***^ (0.016)
Control variable	YES	YES	YES
Year fixed e	YES	YES	YES
Urban fixed effect	YES	YES	YES
Number of observations	5,657	5,502	5,540
*R*-squared	0.068	0.042	0.042

***, **, and * represent significant at the 1, 5, and 10% levels, respectively, and robust standard errors clustered to the city level are in parentheses.

#### 4.4.2 Impact of different types of transfers on public health service levels

The impact of different types of transfer programs on public health service levels may vary, as the targeting and program coverage of different types of transfers differ. Therefore, the welfare impacts of different types of transfers are considered here. Columns (2)–(3) in Table 8 show the impacts of general transfers (*Lngtrans*) and specialized transfers (*Lnstrans*) on public health service levels. Both types contribute to improving welfare, but the coefficient of general transfers is smaller and only significant at the 10% level, while the coefficient of specialized transfers is larger and more significant. This suggests that specialized transfers contribute more to public health service levels than general transfers.

On one hand, this difference may be due to the distinct use and focus of funds between the two types of transfers. General transfers typically provide flexible financial support to local governments, with a broader range of uses, and are not specialized for a particular area. Therefore, their impact on public health service levels may be relatively smaller. In contrast, specialized transfers are financial support for specific areas or programs and may be more focused on improving targeted public health services. Additionally, central specialized transfers to the central and western regions for livelihood expenditures play a key role in compensating and protecting the bottom line of local fiscal expenditures (22), making their impact on welfare more significant.

On the other hand, the difference may also result from the policy objectives and methods of evaluating the effects of the two types of transfers. The effects of general transfers may be broader but more challenging to quantify directly, as they may improve public services indirectly by increasing the overall fiscal revenues of local governments, which might result in a relatively smaller impact on welfare. Specialized transfers are more specifically targeted and may be used directly to provide health care facilities, train medical staff, or improve healthcare coverage for specific groups of people. Consequently, their impact on public health service levels may be more direct and significant.

#### 4.4.3 Impact of transfers on the level of public health under different levels of fiscal self-sufficiency

Fiscal self-sufficiency refers to a local government’s ability to finance its expenditures through its own fiscal revenues. Variations in fiscal self-sufficiency across regions can influence the impact of transfers on educational welfare. This section divides regions into five groups based on their fiscal self-sufficiency, ranging from low to high.

The sub-sample regressions 1–5 in Table 9 show that transfers increase public health services in regions with fiscal self-sufficiency below 0.6. This is likely because local governments in these areas depend on transfers to address fiscal gaps, including in public health. In this context, the impact of transfers on public health may be more pronounced. The effect of transfers on regions with fiscal self-sufficiency between 0.6 and 0.8, and between 0.8 and 1, is either insignificant or negative. This suggests that local governments in regions with higher fiscal self-sufficiency may have more financial resources available for public health. As a result, the impact of transfers on these regions may be relatively small, as local governments have greater autonomy in allocating financial resources. In such cases, transfers may constitute only a portion of fiscal spending, with other revenue sources (e.g., taxes) playing a more significant role, thereby reducing the impact of transfers.

**Table 9:** Impact of transfers on the level of public health under different levels of fiscal self-sufficiency.

Variable	Heal				
	(1) 0–0.2	(2) 0.2–0.4	(3) 0.4–0.6	(4) 0.6–0.8	(5) 0.8–1
*lntran*	0.022^***^ (0.007)	0.050^**^ (0.006)	0.012^**^ (0.005)	0.008 (0.008)	−0.010^*^ (0.006)
*_cons*	0.239^***^ (0.038)	0.286^***^ (0.041)	0.207^***^ (0.036)	0.131^***^ (0.044)	0.300^***^ (0.052)
Control variable	YES	YES	YES	YES	YES
Year fixed effects	YES	YES	YES	YES	YES
Urban Fixed Effect	YES	YES	YES	YES	YES
Number of observations	1,143	1,144	1,144	1,144	1,081
*R*-squared	0.025	0.083	0.050	0.144	0.107

*, ** and *** represent significant at the 10 ,5 and 1% levels, respectively, and robust standard errors clustered to the city level are in parentheses.

## 5 Impact on health inequalities in the residents

The empirical findings of this paper indicate that transfer payments enhance the level of residents’ public health services. However, in reality, transfer payments also serve the function of balancing regional disparities and promoting the equalization of public services. Inequality in public health services is not only linked to the welfare of residents but also affects social fairness and stability within a country or region. Therefore, this paper further explores the impact of transfer payments from the perspective of public health service inequality. The main model forms are as follows:


(5) $$\mathrm{Healt}h_{\mathrm{it}}=\beta_{0}+\beta_{1}\mathrm{Lntra}n_{\mathrm{it}}+\beta_{2}X_{\mathrm{it}}+\alpha_{i}+\mu_{\mathrm{pt}}+\varepsilon_{\mathrm{it}}$$

In Equation 5, $\mathrm{Healt}h_{\mathrm{it}}$ denotes the degree of public health service inequality in city i in year t . Based on the level of public health services, this paper draws on Tsui (23), Abul Naga and Geoffard (24), and Brambilla and Peluso (25), utilizing the Tsui index to measure public health service inequality. The remaining variables are defined as in Equation 1.

The regression results of transfer payments on inequality in public health services are presented in Table 10. Columns (1)–(5) show the results of regressions with different control settings: no control variables or fixed effects, adding control variables only, controlling for individual and year effects only, adding control variables and controlling for individual and year fixed effects, and adding control variables with province and year interaction fixed effects, respectively. Comparing the results across these five columns, it is found that all fiscal transfers have a significant negative effect on public health service inequality, indicating that transfers help reduce inequality in public health services for residents. This suggests that the role of transfers in enhancing public health services aligns with their purpose of promoting the equalization of public services and helps to reduce overall inequality in health services.

**Table 10:** Impact of transfer payments on inequalities in public health services.

Variable	Health				
	(1)	(2)	(3)	(4)	(5)
*Lntran*	−0.015^***^ (0.002)	−0.013^***^ (0.003)	−0.015^***^ (0.005)	−0.014^***^ (0.005)	−0.013^***^ (0.004)
*_cons*	0.190^***^ (0.016)	0.198^***^ (0.018)	0.189^***^ (0.029)	0.202^***^ (0.034)	0.187^***^ (0.033)
Control variable	NO	YES	NO	YES	YES
Year fixed effects	NO	NO	YES	YES	YES
Urban fixed effect	NO	NO	YES	YES	NO
Province × Year fixed effects	NO	NO	NO	NO	YES
Number of observations	5,639	5,638	5,639	5,638	5,638
*R*-squared	0.075	0.076	0.079	0.080	0.079

*** stands for significant at the 1% level, and robust standard errors clustered to the city level are in parentheses.

Huang (26) argues that improving the level of public service equalization is an effective way to alleviate health inequality. Therefore, building on the exploration of the impact of transfer payments on regional inequality in public health services, this section further investigates whether public health services reach micro-individuals within the population and whether fiscal transfers ultimately affect health disparities among individuals. To answer this question, this paper utilizes data from the China Health and Retirement Longitudinal Study (CHARLS) for the years 2011, 2013, 2015, 2018, and 2020. The database provides information on the city to which each sample belongs, enabling the measurement of the health concentration index for sample cities.

Since self-assessed health status is an individual’s comprehensive judgment based on their objective health condition, which can comprehensively reflect the multidimensional aspects of health (27), this section uses residents’ self-assessed health status as a representative indicator of health. The methodology of Wagstaff et al. (28) is used to measure the degree of residents’ health inequality at the micro level through a concentration index. Additionally, since self-assessed health status is an ordered categorical variable with five levels—“poor,” “fair,” “good,” “very good,” and “excellent”—that cannot be directly used to calculate the concentration index, we follow van Doorslaer and Jones (29) and Fan et al. (30) by using an ordered probit model to transform self-assessed health levels into continuous values in the range [0, 1]. The Health Concentration Index (HCI) was then calculated using the following formula:


(6) $$HCI=\frac{2}{M}cov\left(Sah_{i}，R_{i}\right)=\frac{2}{nM}\overset{n}{\underset{i=1}{\sum }}Sah_{i}R_{i}-1$$

In Equation 6. HCI represents the health concentration index, which ranges from −1 to 1. M is the average health level of the sample, and $Sah_{i}$ represents the self-assessed health status in the sample. It is a positive indicator, where higher values indicate better health conditions. $R_{i}$ is the income rank of each individual in the sample (sorted from lowest to highest income). When HCI is positive, it indicates that individuals with higher incomes have better health, reflecting health inequality favoring the rich. Conversely, a negative HCI indicates that it is favorable to the poor.

Table 11 presents the results of the impact of fiscal transfers on residents’ health inequality. Columns (1) and (2) show the effects of transfers on residents’ health inequality and health levels, respectively. The results indicate that fiscal transfers help improve population health and alleviate health inequality among residents. The impact of transfer payments on inequality in the public service dimension has already been discussed in the previous section. Column (3) further examines the effect of inequality in public health services on the health status of individuals. The results show that inequality in public health services is significantly negatively correlated with residents’ health levels, implying that reducing inequality in health services helps improve residents’ health.

**Table 11:** Impact of transfer payments on population health inequalities.

Variable	HCI	Heal	
	(1)	(2)	(3)
*Lntran*	−0.031^**^ (0.015)	0.014^***^ (0.005)	
*Health*			−0.696^***^ (0.070)
Control variable	YES	YES	YES
Year fixed effects	YES	YES	YES
City fixed effect	YES	YES	YES
Number of observations	580	580	580
*R*-squared	0.117	0.163	0.177

** and *** represent significant at the 5 and 1% levels, respectively, and robust standard errors clustered to the city level are in parentheses.

## 6 Possible channels

In this section, we explore three potential channels that could elucidate the causal effects of increased transfers on improved public health services. Increased transfer payments can enhance the financial resources of local governments, which may lead them to increase productive investments in infrastructure, thereby boosting the region’s productivity and competitiveness. This may indirectly improve healthcare infrastructure and enhance the accessibility of healthcare services, contributing to the development of public health services. However, the realization of this indirect effect depends on whether local governments prioritize short-term economic growth or long-term well-being. If they focus more on short-term growth, they may neglect investment in public health services. Therefore, setting clear indicators and assessment standards to strengthen the central government’s responsibility to local governments in improving residents’ comprehensive welfare could help ensure that transfers are utilized effectively for enhancing public health services. Existing research suggests that transfer payments may prompt local governments to alter their fiscal spending priorities to foster regional economic development and maintain fiscal stability (31).

The second potential channel involves the efficiency of fiscal health expenditures, which may improve with increased fiscal transfers. Fiscal transfers can enhance local government finances, enabling them to allocate more resources to health expenditures, thereby increasing welfare investment and improving public health services. Furthermore, the allocation of transfers can guide local governments to direct resources to areas of greatest need, optimizing resource allocation and enhancing the efficiency of fiscal health expenditures. However, the budgeting and execution process for special transfer payments is often neither standardized nor transparent, which grants considerable discretionary power to government departments in charge of special funds. This results in large-scale “running money into the ministries” behavior by local governments, leading to numerous gray transactions and official corruption, which significantly undermines the efficiency of fiscal expenditures (3). Therefore, we propose that another mechanism by which fiscal transfers may influence the level of public health services is through fiscal expenditure efficiency.

The third path of influence is the fiscal gap. In China, due to significant disparities in the level of economic development between regions, transfer payments play a crucial role in redistributing financial resources across regions, enhancing local governments’ ability to provide public services, and promoting the equalization of public services. General transfer payments aim to reduce the financial disparity among local governments and balance their fiscal resources, enabling relatively weaker regions to access more financial resources, which directly increases their capacity to provide basic public services, such as public health services. Special transfers are designed to support specific public projects or services and can specifically target local government spending on public health, disease prevention and control, and medical infrastructure construction, thereby improving the quality and coverage of public health services in these areas.

In this section, empirical tests are conducted using panel data from 283 prefecture-level and above cities from 2001 to 2020. The results are presented in Table 12. Columns (1)–(3) show the regression results of transfer payments on each mechanism variable. Transfer payments (Lntran) are significantly and positively related to fiscal expenditure bias (exp), indicating that as transfer payments increase, local governments are more inclined to allocate these additional funds to welfare expenditures after increasing their financial resources. Transfer payments are also significantly and positively associated with fiscal health expenditure efficiency (heff), suggesting that an increase in transfer payments helps improve the fiscal health expenditure efficiency of local governments. Furthermore, transfer payments (Lntran) are significantly and positively correlated with the fiscal gap (fgap), indicating that transfer payments widen the fiscal gap among local governments. This finding aligns with Wang and Tao (10) conclusion that increased transfer payments exacerbate vertical financial inequality between different levels of government. This may be due to the fact that inter-regional fiscal revenue inequality is still expanding rapidly, and the equalizing effect of fiscal transfers is not sufficient to keep pace with the expanding fiscal revenue inequality, resulting in the continued growth of inter-regional fiscal expenditure inequality.

**Table 12:** Results of mechanistic tests of transfer payments on public health service levels.

Variable	exp	heff	fgap	Heal		
	(1)	(2)	(3)	(4)	(5)	(6)
*Lntran*	0.050^***^ (0.018)	0.065^***^ (0.007)	0.087^***^ (0.023)			
*exp*				0.013^***^ (0.004)		
*heff*					0.114^**^ (0.051)	
*fgap*						0.005 (0.012)
*_cons*	0.297^***^ (0.075)	−0.132^***^ (0.044)	−0.665^***^ (0.158)	0.297^***^ (0.026)	0.318^***^ (0.065)	0.391^***^ (0.009)
Control variable	YES	YES	YES	YES	YES	YES
City fixed effect	YES	YES	YES	YES	YES	YES
Province × Year fixed effects	YES	YES	YES	YES	YES	YES
Number of observations	5,660	5,660	5,660	5,660	5,660	5,660
*R*-squared	0.256	0.809	0.064	0.049	0.051	0.044

*** and ** represent significant at the 1 and 5% level, respectively, and robust standard errors clustered to the city level are in parentheses.

Columns (4)–(6) present the effects of the three mechanism variables on the level of public health services for residents. The coefficients of fiscal expenditure bias (exp) and the level of public health services (heal) are significantly positive, suggesting that fiscal expenditure bias induced by transfers enhances public health service levels. The coefficients of fiscal health expenditure efficiency and the level of public health services (heal) are also positive and significant at the 5% level, indicating that increasing the efficiency of fiscal health expenditure helps narrow the gap in public health services and improve residents’ well-being. The regression coefficient of the fiscal gap (fgap) and the level of public health services (heal) is not significant, implying that statistically, the fiscal gap does not have a significant impact on the welfare of public health services.

Since the results of the benchmark regression indicate that transfer payments significantly improve the level of public health services for residents, combined with the results of the mechanism test, it can be inferred that the efficiency of local governments’ public health expenditures is the primary transmission pathway through which transfer payments influence public health service levels. This suggests that fiscal transfers help optimize resource allocation, improve the efficiency of fiscal health expenditures, and increase local government investment in welfare, thereby enhancing the allocation, quality, and coverage of public health services across regions, ultimately promoting improvements in public health services.

## 7 Conclusion

The quality and accessibility of public health services are essential to the overall health and well-being of society. Understanding and improving the state of public health services amid unequal economic development and disparities in financial resource distribution across regions has become a key challenge for policymakers. Transfer payments, as a form of intergovernmental fiscal allocation, play a vital role in balancing economic development and resource allocation among regions. Investigating how transfer payments affect public health service levels can provide valuable insights for formulating more effective health policies, which is crucial for enhancing the efficiency and equity of public health services and promoting the overall health and well-being of society.

Therefore, this paper constructs an evaluation system for public health service indicators from the perspectives of function and capacity, measuring the level of public health services by combining structural equation modeling and the fuzzy evaluation method. The analysis uses data from 1,871 districts and counties between 2001 and 2020, and measures inequality in the function and capacity of public health services across regions in China using the Tsui index. By employing a fixed effects model, panel threshold effect model, instrumental variable approach, and quantile regression, this study explores the impact of transfer payments on the level of public health services at an empirical level, further analyzing the impact mechanisms and heterogeneous effects of transfer payments. Based on the relevant theoretical and empirical analysis, this paper presents the following main findings:

Consistent with the findings of Guo and Jia (5), we find that fiscal transfers help raise the level of public health services for residents, with no evidence of a non-linear relationship between the two. At the 1% significance level, a 1% increase in fiscal transfers results in a 0.007% increase in the level of public health services. This conclusion remains robust after a series of robustness tests. From a policy perspective, this finding highlights the need for targeted and efficient allocation of transfer payments, focusing on regions or sectors with the greatest need for improvement in public health services.

Distinguishing ourselves from the existing literature, we further examine how transfers affect the level of public health services and find that transfers primarily influence public health service levels through fiscal expenditure bias and fiscal health expenditure efficiency. As transfers increase, both the fiscal expenditure bias of local governments and the efficiency of fiscal health expenditures effectively contribute to raising public health service levels.

In addition, we find heterogeneity in the impact of transfers on public health service levels. Regarding different types of transfers, both general and specialized transfers contribute to improving public health service levels, but specialized transfers are more effective compared to general transfers. Jia et al. (6) also concluded that specialized transfers are most effective in achieving the policy objective of equalizing basic public services. From a regional perspective, the impact of fiscal transfers on public health services in the eastern region is not significant, whereas transfers have a significant positive impact on public health services in the central and western regions. This finding aligns with the results of Tian and Qi (7). The eastern regions of China are economically developed with relatively advanced public health systems. As a result, the marginal benefits of fiscal transfers may be lower in these regions compared to central and western China, where public health infrastructure is less developed and funding needs are more pressing.

Finally, we find that transfer payments not only help reduce inequality in public health services but also improve population health and mitigate health inequality at the micro level. A 1% increase in transfer payments results in a 0.013% decrease in public health service inequality. Fiscal transfers are significantly positively correlated with residents’ health levels and significantly negatively correlated with health inequality. The empirical results suggest that transfer payments help enhance residents’ health levels and alleviate health inequality. Furthermore, the reduction in inequality in public health services is found to contribute to improving residents’ health, indicating that fiscal transfers ultimately enhance health at the micro level by reducing inequalities in public health services.

Based on the findings of this paper, it may be necessary to focus on optimizing transfer payment strategies in the future, tailoring them to the specific conditions of different regions to more effectively reduce inequalities in public health services. Additionally, attention should be given to the efficiency and transparency of fiscal spending, particularly health spending, to ensure resources are used more effectively to enhance public health services. Specifically:

First, increase special transfers to less developed regions. Particularly in the central and western regions, it is recommended to enhance financial support, prioritize improvements in medical infrastructure, and increase investment in medical equipment and personnel.

Second, optimize the transfer payment structure. Increase targeted support for public health service areas, and introduce a performance evaluation mechanism to improve the efficiency of fund usage, ensuring that transfers contribute to better population health. Achieving a balance between specialized and general transfers to ensure a more targeted and efficient allocation of resources across regions, while also balancing equity and efficiency.

Third, enhance local governments’ management capacity. Fiscal transfers may be less effective in regions with inefficient governance, as funds may not be allocated or utilized optimally. Through financial incentives and training, local governments should be empowered to manage public services more effectively, ensuring that health funds are utilized in the most efficient manner. The design of fiscal transfer programs should incorporate performance-based incentives to ensure that transfers are tied to measurable improvements in public service delivery.

## Data Availability

Publicly available datasets were analyzed in this study. The basic data used in this paper comes from the open data repository of Peking University. The DOI URL is: https://doi.org/10.18170/DVN/45LCSO.
